# Computational identification of variables in neonatal vocalizations predictive for postpubertal social behaviors in a mouse model of 16p11.2 deletion

**DOI:** 10.1038/s41380-021-01089-y

**Published:** 2021-04-15

**Authors:** Mitsuteru Nakamura, Kenny Ye, Mariel Barbachan e Silva, Takahira Yamauchi, Daniel J. Hoeppner, Amir Fayyazuddin, Gina Kang, Emi A. Yuda, Masako Nagashima, Shingo Enomoto, Takeshi Hiramoto, Richard Sharp, Itaru Kaneko, Katsunori Tajinda, Megumi Adachi, Takuma Mihara, Shinichi Tokuno, Mark A. Geyer, Pilib Ó Broin, Mitsuyuki Matsumoto, Noboru Hiroi

**Affiliations:** 1 Department of Pharmacology, University of Texas Health Science Center at San Antonio, San Antonio, TX, USA; 2 Department of Bioengineering, Graduate School of Engineering, The University of Tokyo, Tokyo, Japan; 3 Department of Epidemiology and Health Science, Albert Einstein College of Medicine, Bronx, NY, USA; 4 School of Mathematics, Statistics & Applied Mathematics, National University of Ireland Galway, Galway, Ireland; 5 La Jolla Laboratory, Astellas Research Institute of America LLC, San Diego, CA, USA; 6 Graduate School of Engineering, Tohoku University, Sendai, Japan; 7 Department of Psychiatry, Albert Einstein College of Medicine, Bronx, NY, USA; 8 Department of Psychiatry, University of California, San Diego, La Jolla, CA, USA; 9 Graduate School of Medical Sciences, Nagoya City University, Nagoya, Japan; 10 Drug Discovery Research, Astellas Pharma Inc., Ibaraki, Japan; 11 Graduate School of Health Innovation, Kanagawa University of Human Services, Kawasaki-shi, Kanagawa, Japan; 12 Department of Cellular and Integrative Physiology, University of Texas Health Science Center at San Antonio, San Antonio, TX, USA; 13 Department of Cell Systems Anatomy, University of Texas Health Science Center at San Antonio, San Antonio, TX, USA; 14 Department of Psychiatry, University of Texas Health Science Center at San Antonio, San Antonio, TX, USA

## Abstract

Autism spectrum disorder (ASD) is often signaled by atypical cries during infancy. Copy number variants (CNVs) provide genetically identifiable cases of ASD, but how early atypical cries predict a later onset of ASD among CNV carriers is not understood in humans. Genetic mouse models of CNVs have provided a reliable tool to experimentally isolate the impact of CNVs and identify early predictors for later abnormalities in behaviors relevant to ASD. However, many technical issues have confounded the phenotypic characterization of such mouse models, including systematically biased genetic backgrounds and weak or absent behavioral phenotypes. To address these issues, we developed a coisogenic mouse model of human proximal 16p11.2 hemizygous deletion and applied computational approaches to identify hidden variables within neonatal vocalizations that have predictive power for postpubertal dimensions relevant to ASD. After variables of neonatal vocalizations were selected by least absolute shrinkage and selection operator (Lasso), random forest, and Markov model, regression models were constructed to predict postpubertal dimensions relevant to ASD. While the average scores of many standard behavioral assays designed to model dimensions did not differentiate a model of 16p11.2 hemizygous deletion and wild-type littermates, specific call types and call sequences of neonatal vocalizations predicted individual variability of postpubertal reciprocal social interaction and olfactory responses to a social cue in a genotype-specific manner. Deep-phenotyping and computational analyses identified hidden variables within neonatal social communication that are predictive of postpubertal behaviors.

## Introduction

Because copy number variants (CNVs) are robustly associated with autism spectrum disorder (ASD) and other developmental neuropsychiatric disorders, such genetic variants provide a genetically homogeneous and identifiable entry point toward a better understanding of psychiatric disorders [1-3]. However, highly variable developmental trajectories remain a major challenge. Not all CNV carriers are diagnosed with developmental psychiatric disorders (i.e., incomplete penetrance), and the precise disorder and their symptomatic severity vary considerably (i.e., variable expressivity).

Mouse models of CNVs—despite caveats concerning differences among species—allow the impact of gene dosage on behavioral dimensions to be experimentally manipulated and isolated from extraneous variables. Moreover, mouse models provide a technical means to prospectively identify, within 1 month, neonatal signs that predict later behavioral abnormalities, which require a much longer period for humans. Many CNV mouse models with well-controlled genetic backgrounds (e.g., 1q21.1, 3q29, 15q11.13, maternal 15q11-13, 16p11.2, and 22q11.2) often do not exhibit deficits in social behaviors and other behavioral dimensions relevant to developmental neuropsychiatric disorders, however [4-12].

Among early predictors, high-pitched cries during infancy are the earliest incipient sign of later diagnosis of idiopathic cases of ASD in humans [13-15]. In mice, atypical neonatal vocalizations of pups with an ASD risk gene variant do not elicit optimal maternal behaviors [16] and are considered an integral deficit of social communication [13, 17]. However, due to weak or absent deficits in social behaviors in mouse models of CNV, it has been difficult to determine the predictive value of neonatal vocalization for later social behaviors.

Carriers of hemizygous deletion at human chromosome 16p11.2 exhibit many developmental neuropsychiatric disorders [18, 19]; approximately one-fifths of 16p11.2 deletion carriers are diagnosed with ASD [19]. Children with 16p11.2 deletion variably show atypical developmental trajectories of motor, social, and cognitive dimensions [20]. Arbogast et al. [21] generated the first coisogenic mouse model of 16p11.2 deletion, in which ES cells derived from C57BL/6N mice were used for gene targeting and the same inbred mouse line was used as a breeder. This study elegantly addressed the interpretative limitation of earlier pioneering work that used noncongenic models. Like other coisogenic and congenic CNV mouse models [6, 12], this coisogenic model exhibited no abnormality in social interaction or preference. The genuine contribution of this and other CNVs to phenotypes relevant to ASD and link between neonatal signs and later phenotypes remains unclear.

As many extraneous variables in testing environments mask or exaggerate phenotypes in mice [22, 23], we developed an independent line of coisogenic mouse model of proximal 16p11.2 hemizygous deletion. Our deep-phenotyping and computational approaches identified call types and call sequences of neonatal social communication that predict postpubertal social behaviors in a genotype-dependent manner even in this largely asymptomatic model. Our approaches provide a technical means to determine the developmental trajectories with variables within dimensions of developmental psychiatric disorders.

## Methods and materials

The actual experiments were conducted while the Noboru Hiroi was at Albert Einstein College of Medicine. Animal handling and use followed the protocols that were approved by the Animal Care and Use Committees of Albert Einstein College of Medicine, in accordance with NIH guidelines. The analysis and manuscript were completed at the University of Texas Health Science Center at San Antonio where the Noboru Hiroi is currently employed.

We developed a coisogenic mouse model of 16p11.2 deletion (see Supplementary Information (SI), Mouse and RNA-seq). We recorded and analyzed their behaviors during the neonatal period (P8 and P12) [24], and during the postpubertal period starting at the age of 1 month (see SI, Behavioral Analyses). The minimal sample size was determined by power analyses based on our previous study [16]. Data were computationally analyzed using the Lasso regression model, random forest, Markov model, and linear regression model (see SI, Computational Analyses).

We compared group means using analysis of variance, followed by Newman–Keuls post hoc tests, if interaction was significant. Two-sided *t*-tests were used when there were only two groups. A probability of ≤0.05 was considered significant. When multiple tests were applied to a dataset, the significance level was adjusted using Benjamini–Hochberg’s correction. When either the assumption homogeneity of variance or normality was violated, data with a repeated measure were analyzed by a generalized linear mixed model; for comparisons of a pair of data, nonparametric tests were used.

## Results

### Coisogenic mouse model of 16p11.2 deletion

We developed a coisogenic mouse model of human proximal 16p11.2 hemizygous deletion through in vitro Cre-mediated recombination of a 378 kb region of the 7qF3 region spanning from *Mapk3* to *Spn* genes (Fig. S1A). RNA-seq analysis confirmed that the expression of genes encoded in the deleted region was reduced in the targeted region (Fig. S1B). However, reduction of some genes did not reach statistical significance in some brain regions due to large variance (e.g., *Tbx6, Pagr1a, Pagr1b,* and *Zg16*) or very low baseline expression (i.e., floor effect).

### Characterization of neonatal vocalizations and postpubertal social behaviors

As each genetic variant is likely to cause unique—as well as common—phenotypic features, finding a phenotype is fundamentally exploratory and does not permit a priori hypotheses. Because of this, mice were tested for neonatal vocalizations and then for a wide range of other behavioral dimensions thought to be relevant to ASD and other developmental neuropsychiatric disorders, at 1 month of age, when mice start to show early signs of puberty [25].

**Fig. 1:**
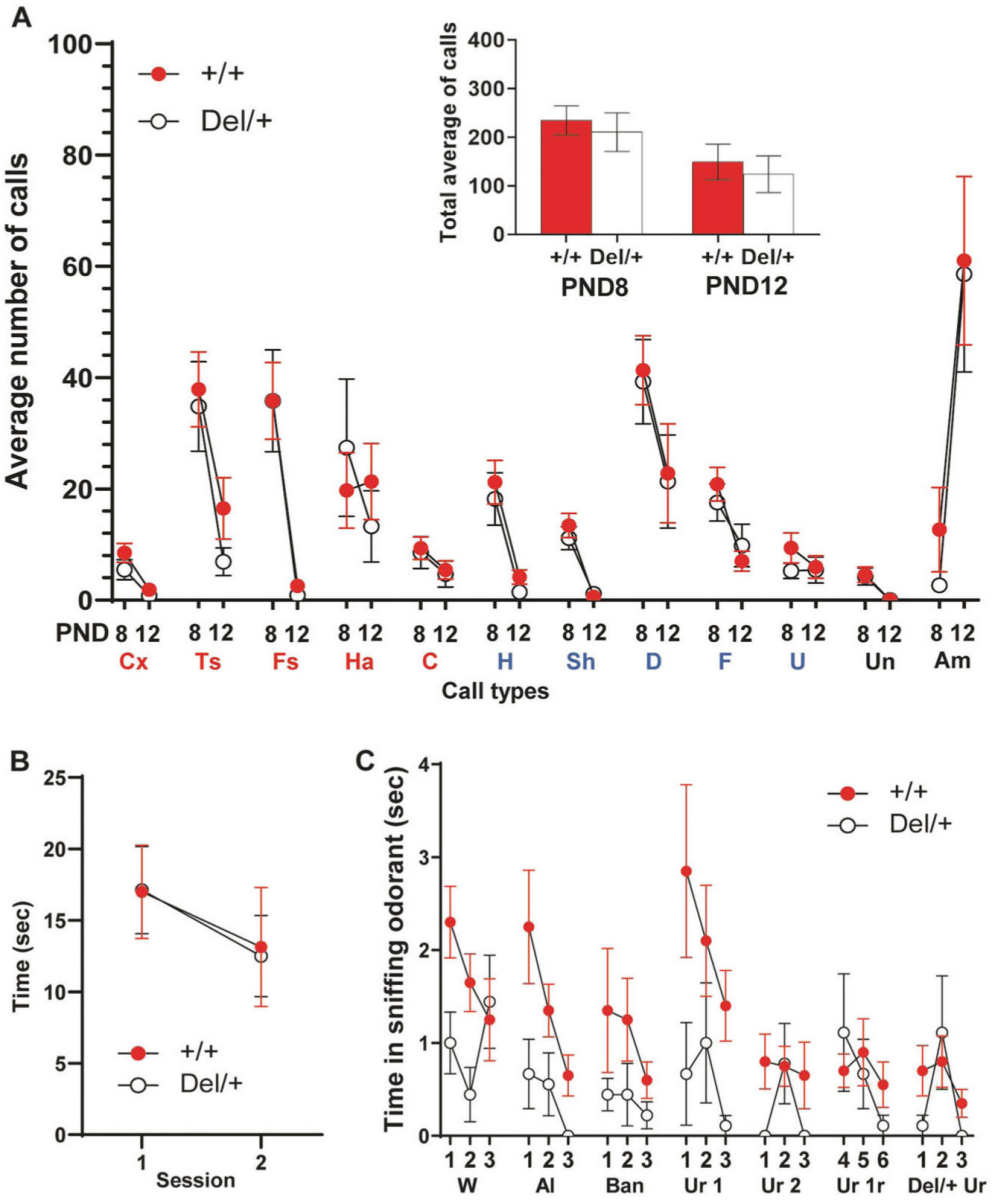
Neonatal and postpubertal social behaviors. **A** The average (+SEM) is shown. +/+ and Del/+ mice were indistinguishable in the total number of each call type emitted during the 5-min recording period at postnatal days 8 and 12. A mixed model analysis showed no effect of genotype or its interaction with other factors (genotype, *F*(1, 56.285) = 0.085, *p* = 0.771; genotype × age, *F*(1, 1199.982) = 0.204, *p* = 0.652; genotype × call type, *F*(11, 1137.054) = 0.150, *p* = 0.999; genotype × age × call type, *F*(11, 1137.054) = 0.303, *p* = 0.985). Inset: +/+ and Del/+ mice were indistinguishable in total number of calls emitted during the 5-min recording period (Mann–Whitney test, P8, *U* = 386, *p* = 0.5535; P12, *U* = 207, *p* = 0.8026). +/+: P8, *N* = 37; P12, *N* = 29. Del/+, P8, *N* = 23; P12, *N* = 15. Multiple-wave call types (red): Cx complex, Ts two syllable, Fs frequency steps, Ha harmonics, C composite. Simple call waves (blue): H hump, Sh short, D downward, F flat, U upward. Other types (black): A ambiguous, Un uncharacterized. **B** Time (means ± SEM) spent in active, affiliative social interaction is shown in two successive 5-min sessions at 1 month of age (Mann–Whitney test; Session 1, *U* = 50, *p* = 0.7008; Session 2, *U* = 44.5, *p* = 0.4495). +/+, *N* = 14; Del/+, *N* = 8. **C** +/+ mice spent more time (mean(s) ± SEM) in sniffing at an Eppendorf tube containing each odorant at Trial 1 and exhibited a higher degree of habituation to each odorant than Del/+ mice at 1 month of age. As the assumption of normality in all cases except for Del/+, water, Trial 1, and of homogeneity of variance in 11 out of 21 comparisons between +/+ and Del/+ was violated, we used a generalized linear mixed model. +/+ and Del/+ mice differ (genotype, *F*(1, 27) = 8.966, *p* = 0.006; odorant, *F*(6, 540) = 3.795, *p* = 0.001; trial, *F*(2, 540) = 5.747, *p* = 0.003; genotype × odorant, *F*(6, 540) = 1.727, *p* = 0.113; genotype × trial, *F*(2, 40) = 1.221, *p* = 0.296; genotype × odorant × trial, *F*(12, 540) = 0.583, *p* = 0.857). W water, Al almond odorant, Ban banana odorant, Ur 1 urine of one male C57BL/6J mouse, Ur 2 urine of another male C57BL/6J mouse, Ur1r a second exposure to the urine of the first male C57BL/6J mouse (urine 1), DEL/+Ur urine of one male nonlittermate Del/+mouse. +/+, *N* = 20; Del/+, *N* = 9.

Neonatal Del/+ and +/+ littermates were indistinguishable at postnatal (P) day 8 and P12 in the average numbers of each call type (Fig. 1A) and in all call types (Fig. 1A, inset). At the age of 1 month, Del/+ mice and +/+ littermates were indistinguishable in reciprocal social behavior (Fig. 1B). However, Del/+ mice exhibited fewer olfactory responses to both nonsocial and social odorants than +/+ mice (Fig. 1C).

By contrast, Del/+ mice were indistinguishable from +/+ littermates in other standard behavioral assays at 1 month of age: approach to a novel, nonsocial object and its habituation (Fig. S2A), anxiety-related behavior in an elevated plus maze (Fig. S2BC), acoustic startle (Fig. S2D), prepulse inhibition (Fig. S2E), working memory/repetitive behavioral traits in a T-maze (Fig. S2F), motor behavior (Fig. S2G), and thigmotaxis (Fig. S2H). Mice were additionally tested at 2 months for working memory only, as severe working memory deficits often appear later during development in mouse models of genetic variants associated with ASD [26, 27] and humans with ASD [28-34]. Del/+ and +/+ mice were indistinguishable in this task at 2 months of age (data not shown). The phenotypes in olfactory responses (see Fig. 1C) are not due to altered responses to an object or anxiety responses to novel objects, as Del/+ and +/+ mice were indistinguishable in their responses to a novel object (see Fig. S2A) or anxiety-related traits (see Fig. S2B, C, H). Consistent with the previous report of another coisogenic mouse model of 16p11.2 [21], our Del/+ model was significantly underweight compared to +/+ mice throughout development (Fig. S3). However, this physical developmental delay did not impact any neonatal or postpubertal behaviors, except for postpubertal olfactory responses (see Figs. 1A, B and S2A-H).

### Computational extraction of predictive variables of neonatal vocalizations for postpubertal social behaviors

We ran least absolute shrinkage and selection operator (Lasso) regression model on the data to extract predictive features (Fig. S4, predictive model, Lasso regression model 1), as it is ideal to extract a small number of most robust predictive features from a large pool of collinear parameters when the number of variables is greater than the number of observations. The candidate explanatory variables were genotype, acoustic parameters, number and ratios of distinct call types, and number and probabilities of distinct call transitions (i.e., sequences). The communicative capacity of neonatal vocalization sequences and their relevance to ASD have been demonstrated in a mouse model of a genetic variant linked to developmental neuropsychiatric disorders [16, 35]. The dependent variables were social interaction at Session 1, its habituation from the first to last session (i.e., Session 1 – Session 2), olfactory responses to the urine smell at Trial 1, and its habituation from the first to last trial (i.e., Trial 1–Trial 3).

**Fig. 2:**
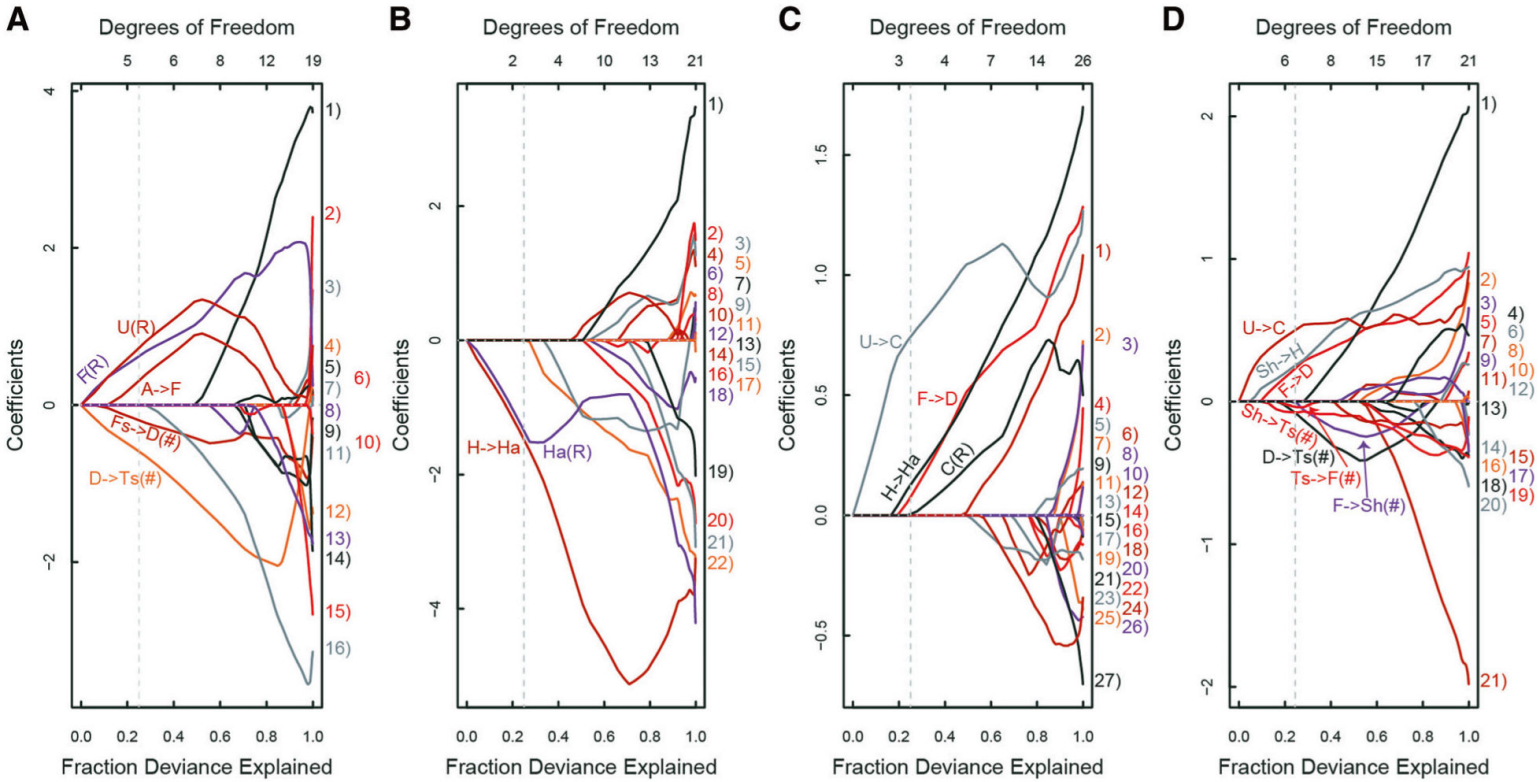
Fraction deviance explained and coefficients of variables determined by Lasso regression model 1 (see Fig. S4). All parameters (call type number and ratios, call sequence number and probabilities, acoustic parameters) were used for selection. **A** Social interaction. **B** Social habituation (Session 1 score – Session 2 score). **C** Olfactory response. **D** Olfactory habituation (Trial 1 score – Trial 3 score). Cutoff was set at 0.25 of fraction deviance explained (see light gray vertical broken line). The probabilities or number (#) of two-call sequences and ratio (R) of the number of a call type emitted to all calls emitted were selected (see Table S1 for detailed labels). Lasso extracts features along the proportion of deviance explained. The regularization parameter lambda decreases as the proportion of deviance explained increases. Thus, large lambda values (i.e., small values along the axis of the proportion of deviance explained) are robust predictors. Cx complex, Ts two syllable, Fs frequency steps, Ha harmonics, C composite. H hump, Sh short, D downward, F flat, U upward. Other types: A ambiguous, Un uncharacterized. The number of mice that completed social interaction or olfactory response testing and emitted more than ten calls at P8 were +/+, *N* = 11; Del/+, *N* = 8 for social interaction and +/+, *N* = 16; Del/+, *N* = 8 for olfactory response.

Each postpubertal social behavior had its unique predictive signature of neonatal calls (Fig. 2; Table S1). Many selected predictors were the transition probabilities and numbers of transitions from one call to another (e.g., Fs→D (#) and A→F for social interaction; H→Ha for social habituation; U→C for olfactory response and olfactory habituation) (Table S2). The ratios of some call types also were selected (e.g., U(R) for social interaction; Ha(R) for social habituation; C(R) for olfactory response). The identified call features had good predictive values for the four readouts of behaviors of +/+ mice, but not Del/+ mice (see Table S2).

One possibility for this observation is that neonatal call features predictive of postpubertal social behaviors are less robust in Del/+ than in +/+ mice. To find neonatal call features that are predictive of postpubertal social behaviors in Del/+ mice, we carried out another Lasso regression with genotype as the dependent variable (Fig. S4, Lasso regression model 2). This model selected a unique set of call features as predictors (Fig. S5). Random forest, with Del/+ as a positive reference dependent variable, showed high specificity (0.7917) (Table S3), indicating that the genotype +/+ can be fairly accurately identified by certain call features (i.e., a low false positive rate). In contrast, the predictors had low sensitivity (0.5000) and sensitivity and specificity are significantly different (McNemar’s test, *p* = 1.012 × 10^−5^); the selected features often misjudged Del/+ mice as +/+ (i.e., false negative). As a result, the overall accuracy was significant (*p* = 8.837 × 10^−8^) but modest (0.681), suggesting that there are proportionally more neonatal call features predictive of +/+ genotype than Del/+ genotype. Some of the selected features predicted social behaviors in a genotype-dependent manner (Table S4).

**Fig. 3:**
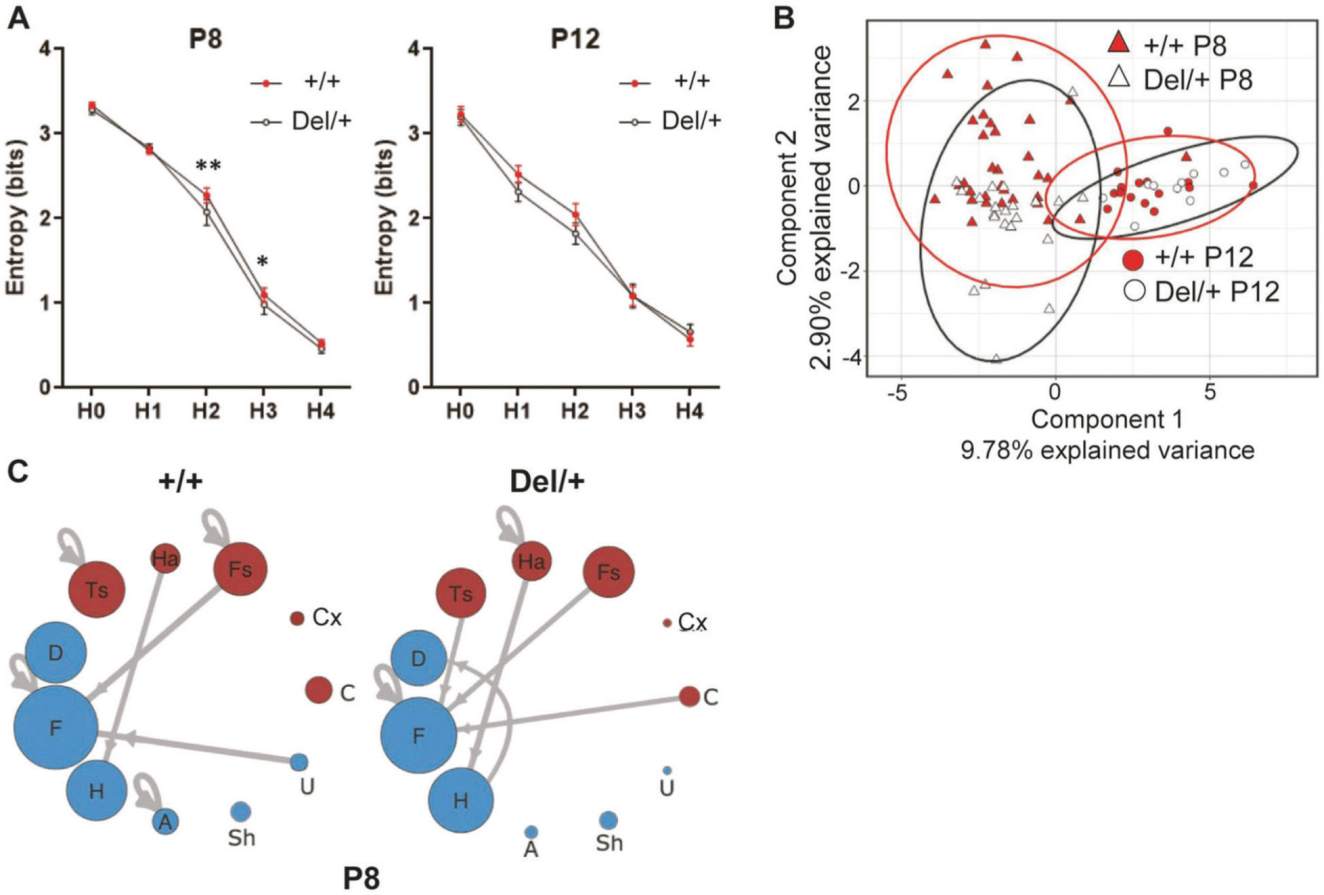
Sequence structures of neonatal calls. **A** Shannon entropy analysis of the randomness of call repertoires (H0), of individual calls used within the repertoire (H1), of two-call sequence (H2), three-call sequence (H3), and four-call sequence (H4). +/+ mice and Del/+ mice differed at H2 (*p* = 0.00761) and H3 (*p* = 0.04467) at P8, as determined by linear mixed model fitted using REML with *t*-test degrees of freedom approximated using Satterthwaite’s method. *p < 0.05; **p < 0.001. **B** sPLS-DA analysis of call. Two factors segregated two-call sequences in terms of age (Component 1) and, to a lesser extent, genotype of P8 data (Component 2). **C** Markov models identified the most frequent call connections between two calls at P8. Multiple-wave call types (red): Cx complex, Ts two syllable, Fs frequency steps, Ha harmonics, C composite. Simple call waves (blue): H hump, Sh short, D downward, F flat, U upward, A ambiguous, Un uncharacterized. +/+: P8, *N* = 33; P12, *N* = 22. Del/+: P8, *N* = 16; P12, *N* = 10.

The probabilities and numbers of call sequences were extracted as predictors for postpubertal behaviors more often than any other variables (see Fig. 2) and for genotype (see Fig. S5). To further explore call sequences, we used Shannon entropy analysis, sparse partial least squares discriminant analysis (sPLS-DA), and Markov models (see Fig. S4). Shannon entropy analysis determined the degree of randomness in how many call types pups used (H0), how often pups nonrandomly chose call types within their repertoire (H1), or how pups nonrandomly chose call types in two-call (H2), three-call (H3), and four-call sequences (H4) (Fig. 3A). +/+ and Del/+ differed at H2 and H3 of P8, but not at any level of P12.

We next applied sPLS-DA analysis to select variables based on their relative contribution to classification of genotype to examine individual and group-level variability using two-call sequences. Individual mice are plotted in the two-component model space (Fig. 3B). Component 1 separated pups in terms of age, which represented a developmental change in call sequences. In Component 2, the two genotype groups did not well segregate, but more individually separated at P8 than P12, indicating larger individual variability in the use of specific two-call sequences at P8 than at p12.

While the sPLS-DA analysis revealed individual variability in two-call transitions, there might be some call sequences that are frequently emitted by either genotype. We thus applied Markov models to two-call sequences within each genotype. Call sequences frequently emitted by +/+ pups were Ts→Ts, Fs→Fs, U→F, and A→A, while call sequences frequently emitted by Del/+ pups were Ha→Ha, Ts→F, H→D, and C→F; other call sequences (F→F, Ha→H, and Fs→F) were frequently emitted in both +/+ and Del/+ pups (Fig. 3C). Ha→H and H→D had significant model fit as predictors for social interaction and olfactory habituation, respectively, in Del/+ (Table S5).

Our comprehensive feature extraction (see Fig. S4, Lasso regression models 1 and 2) did not detect acoustic parameters as robust predictors except for maximum frequency minimum parameter of the entire call (MaFmi) for genotype (see Fig. S5). Random forest (see Fig. S4, Radom Forest 2) showed that quantitative acoustic features yielded a low level of accuracy (0.5642) with low sensitivity (0.4946) and modest specificity (0.6338) (Table S6) for genotype, confirming that acoustic parameters are not good predictors.

Among all call features extracted from Lasso and linear regression models, there were neonatal call sequences and call types that are positively or negatively correlated with postpubertal social behaviors (Fig. S6). For example, high and low probabilities of neonatal A→F and Fs-D(#), respectively, predict high levels of postpubertal social interaction in +/+ mice; the higher Ha→H is, the higher their postpubertal social interaction is in Del/+ mice (Fig. S6A). H→Ha, Ha(R), and Ha→Ha negatively predicted social habituation in +/+ mice (Fig. S6B). U→C, F→D, and C(R) in +/+ and D→U(#) in Del/+ positively predicted olfactory response (Fig. S6C). U→C and Sh→H in +/+ mice and D→U(#) and H→D in Del/+ mice positively predicted olfactory habituation; D→Ts(#), Sh→Ts(#), and Ts→F(#) negatively predicted olfactory habituation in +/+ mice (Fig. S6D).

**Fig. 4:**
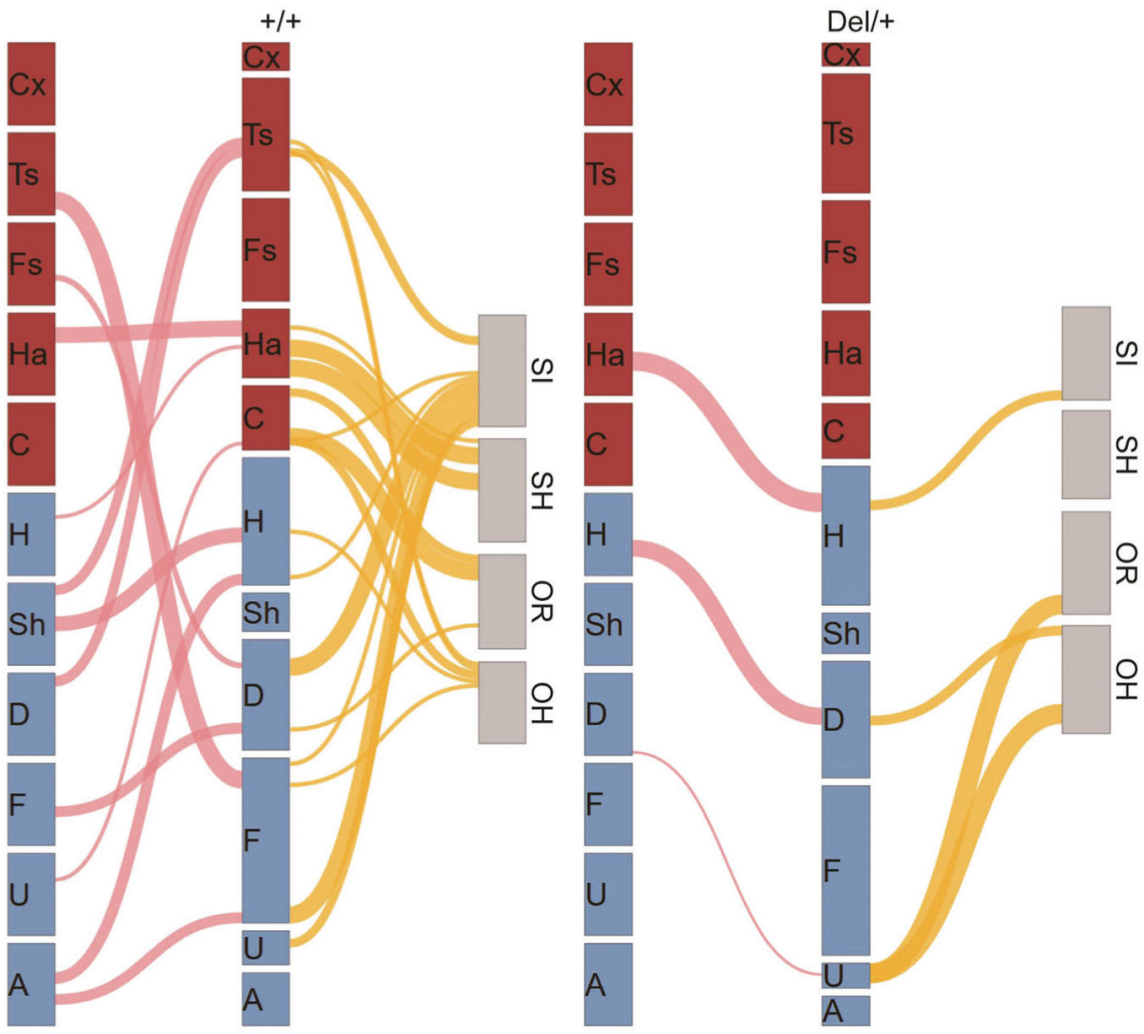
Many paths from neonatal call types and sequences to postpubertal social behaviors. The thickness of each path from one call type to the next represents the proportion of such transition probabilities from one call to another (see pink lines). Only significant correlation coefficients (*p* < 0.05) are shown (see Tables S2, S4, and S5). The strength of paths predicting each postpubertal social behavior is shown as thickness of lines from the second call column to postpubertal social behavior. Only cases in which goodness of fit was significant (Tables S2, S4, and S5) are shown (see yellow lines). The thickness of lines with different levels of significance (*p* < 0.05, *p* < 0.01, and *p* < 0.001) are represented by an arbitrary thickness unit (*x*), 2*x*, and 4*x*, respectively. Cx complex, Ts two syllable, Fs frequency steps, Ha harmonics, C composite, H hump, Sh short, D downward, F flat, U upward. Other types: A ambiguous, Un uncharacterized.

Our analyses collectively identified many paths from specific calls and call sequences to postpubertal social behaviors in a genotype-dependent manner (Fig. 4). Several patterns emerged. First, there is an overall shift of predictors from call types with multiple waves (Fig. 4, red call type bars) to simple call types (Fig. 4, blue call type bars) in Del/+ mice. Second, nonidentical sets of neonatal call features were predictors for the four postpubertal social behavioral readouts (see Figs. 4 and S6). Third, all predictors were genotype-specific within each postpubertal social behavioral readout (see Fig. 4 and Tables S2, S4, and S5). Fourth, some call features predicted more than one postpubertal social behaviors (see Fig. 4 and U→C in Fig. S6A, C, D; D→Ts(#), Fig. S6A, D; D→U(#), Fig. S6C, D).

That call sequences are most frequently identified as predictors among all neonatal call variables further strengthens our hypothesis that ASD risk gene variants have a negative functional impact on social communication between pups and mothers through altered call sequences [16, 35].

## Discussion

Deep-phenotyping analyses showed that our coisogenic mouse model of 16p11.2 deletion generally lacks behavioral phenotypes in standard dimensional measures that are considered relevant to ASD. However, two subdimensional features differentiated genotypes: (1) frequently emitted neonatal call sequences and (2) olfactory responses to nonsocial and social odor. Our computational analyses identified neonatal call types and sequences that differentially predicted individual levels of postpubertal social interaction and olfactory responses to a social cue in a genotype-dependent manner. These data reveal a genotype-specific hidden structure in developmental trajectories from neonatal social communication to postpubertal social behaviors. As many well-controlled mouse models of genetic risk factors show weak or few phenotypic abnormalities in standard measures [6], our approach provides a novel means to identify hidden subdimensions and altered developmental paths in apparently asymptomatic mouse models of not just 16p11.2 CNV but also of many other genetic risk variants for developmental neuropsychiatric disorders.

Our model isolated effects of 16p11.2 deletion against a homogeneous genetic background; therefore, any phenotypic difference can be ascribed to the deletion. Consistent with observations in another coisogenic mouse model of 16p11.2 deletion with a C57BL/6N background tested between 3 and 4 months of age [21], our 16p11.2 deletion model was indistinguishable from +/+ littermates in the total number of calls or of each call type during the neonatal period and prepulse inhibition, reciprocal social interaction, working memory, and repetitive behavioral traits in spontaneous alternation, anxiety-related behaviors in the elevated plus maze, and thigmotaxis and locomotor activity in an open field at the age of 1 month; their coisogenic model with C57BL/6N background was not tested for olfactory responses. While the lack of working memory deficits was consistent with what is seen in individuals with 16p11.2 deletions [36], apparently normal reciprocal social interaction in our and their mouse models is inconsistent with high rates of ASD in 16p11.2 deletion carriers. Although individuals with 16p11.2 deletions have not been characterized for neonatal cries or olfactory response, idiopathic cases of ASD exhibit abnormalities in neonatal cries [13] and olfactory responses [37, 38], but are normal in prepulse inhibition under the standard test condition [39-43].

The general lack of phenotypes in our and their coisogenic mouse models of 16p11.2 deletion could be interpreted as suggesting that developmental neuropsychiatric disorders seen at elevated rates among 16p11.2 deletion carriers are not primarily caused by this chromosomal deletion alone. This CNV might require other coexisting genetic variants, including second CNVs [44] and common genetic variation [21, 45], and environmental insults, such as preterm and C-section birth [46] to be fully symptomatic. Alternatively, this CNV might manifest its impacts on subdimensions that are predominantly used for certain functions in a given species. Our observation underscores the importance of exploratory, deep behavioral phenotyping to identify the phenotypic points within a dimension at which the impacts of genetic variants appear.

Our coisogenic mouse model of 16p11.2 was insensitive to the presentation of new nonsocial and social odorants. This phenotype does not reflect nonspecific sensory or motivational deficits to respond to a social cue, a novel object or anxiety-evoking stimuli (see Fig. 1B; Fig. S2A-C, H). As Del/+ mice are also impaired in their responses to nonsocial odorants (e.g., water), it is still possible, however, that 16p11.2 hemizygosity impairs olfactory sensation, motivation to respond to nonsocial and social olfactory cues, or both. Altered responses to various nonsocial and social olfactory stimuli have been noted in mouse models of other genetic variants associated with ASD [47, 48]. In humans, individuals with idiopathic ASD respond abnormally to both nonsocial and social olfactory stimuli [37, 38, 49]. Individuals with ASD do not differentiate between social and nonsocial odorants [38] and have generally blunted differentiating responses to pleasant and aversive nonsocial odor, and this lack of differentiating response to nonsocial odorants is correlated with their social deficits [37]. Thus, while the exact nature of altered olfactory responses in genetic mouse models of ASD, including ours, remains unclear, defective olfactory responses to both nonsocial and social odorants are a dimension of ASD.

We do not rule out the possibilities that 16p11.2 hemizygosity indirectly impacted neonatal call sequences and olfactory responses via craniofacial abnormalities and a developmental delay in body weights, respectively. However, it is difficult to explain why craniofacial abnormalities did not alter the frequencies and duration of various call types in Del/+ pups and why the developmental delay had no impact on any other social and cognitive behaviors (i.e., social interaction, novel object approach, and working memory). More work is needed to explore the possibilities that altered sequences of neonatal vocalizations and altered olfactory responses are manifestations of a shared physical or neuronal abnormality at different developmental stages.

Our data are inconsistent with reported data of noncongenic models of 16p11.2 deletion. Those models were reported to not differ from their wild-type littermates in olfactory responses to social or nonsocial cues [50-52]. Furthermore, noncongenic models of 16p11.2 deletion were reported to emit more downward and short calls and fewer frequency steps in neonatal vocalizations than their wild-type littermates [51]. Several likely sources of this apparent inconsistency include sex, age of testing, and housing conditions. Another fundamental difference is the degree of homogeneity of genetic background. When mutant mice are not backcrossed to an inbred strain for ten or more generations, noncongenic mutant mice carry, at the chromosomal loci flanking the deleted segment, more alleles of mouse strain of ES cells (e.g., 129SvJ) than those derived from a breeder strain; in contrast, wild-type littermates carry more alleles of a breeder strain (e.g., C57BL/6J) at the same loci than mutant mice. These different genetic backgrounds systematically persist among many cohorts due to a low rate of recombination between the deleted locus and nearby loci. Such a confounding factor results in behavioral, neuronal, cellular, and molecular phenotypic differences that might not be attributable to the mutated gene [6, 53-57]. Use of commercially available B6129SF1/J mice does not serve as a valid control for this confounding factor either, as a mutant mouse is not an F1 generation and still carries more ES cell alleles at the flanking loci than such “control” mice, thereby still creating consistent and systematic differences in the genetic backgrounds between such “wild-type control” and mutant littermates [6]. Moreover, the absence of phenotypic differences in noncongenic models poses an additional interpretative issue, as the baselines of many behavioral and neural phenotypes differ widely among inbred mouse strains [57]. If systematic enrichment of ES cell alleles in mutant mice causes a higher baseline than wild-type mice, any reduction in scores due to the targeted mutation might simply result in an apparently normal phenotype.

Our computational approaches have translational value. Human babies who are later diagnosed with idiopathic ASD (i.e., incipient ASD babies) exhibit atypical cries [13], but the trajectories of dimensions from the neonatal period to later diagnosis of ASD are heterogeneous and unstable [58-61]. Our approach to focus on variables within dimensions, instead of categorical classification of ASD or dimensions per se, could be useful in determining the structure of developmental trajectories in both CNV-associated and idiopathic cases of ASD in humans, as well as in genetic mouse models of CNVs.

## Supplementary Material

Suppl Information

## Data Availability

All data and programs will be provided upon request.
