# Supplementary material for: Computational identification of variables in neonatal vocalizations predictive for postpubertal social behaviors in a mouse model of 16p11.2 deletion
Source: Mol Psychiatry. Author manuscript; Available in PMC 2022 Jan 16. (PMC8517042; doi:10.1038/s41380-021-01089-y)
Supplement: Supplementary file 1 [file NIHMS1698022-supplement-Suppl_Information.docx]

Mitsuteru Nakamura et al

**Supplemental Information**

**Mouse**. We generated mice carrying a microdeletion of the 7qF3 region spanning 378 kb from *Mapk3* gene to *Spn* gene, a mouse region homologous to the human 16p11.2 locus, through *in vitro* Cre-mediated recombination at Taconic Biosciences (**Fig. S1A**). LoxP sites were introduced sequentially upstream of *Mapk3* and *Spn* genes in Taconic Artemis C57BL/6NTac ES cells. Homologous recombination clones were isolated with hygromycin and puromycin selection. The doubly targeted ES clone cells were transfected with a Cre recombinase expression plasmid, and the microdeletion occurs in chromosome with cis arrangement of loxP sites, which were verified by southern blots as well as PCR analysis. These targeted ES cells with the microdeletion were microinjected into blastocysts harvested from BALB/c females and transferred to pseudopregnant NMRI females. Highly chimeric mice as assessed by coat color contribution were bred to Flp-Deleter mice (C57BL/6-Tg(CAG-Flpe)2 Arte, C57BL/6NTac background) to remove puromycin and neomycin section markers. Germline transmission was identified by the presence of black C57BL/6N offspring. The mouse colony was maintained by mating heterozygous 16pDel/+ males with C57BL/6NTac females (Taconic, Rensselaer, NY), to control for the two genotypes’ genetic background^1^. Genotypes for +/+ and Del/+ alleles were identified by PCR using the following primers: For +/+ 5’-CCA TTG TTA GCA GAC GGT AGC-3’ and 5’-GCA AGT CTA GAG ATG CAG AGA CC-3’. For Del/+ 5’-CTA TGG TTC CAA GAC AGT GTG G-3’ and 5’-TCA TCC TCA GAT ACA GAT TGA GC-3. Mice were weaned and separated by sex at P21. We used male mice, as some ASD elements are more severe in male than female 16p11.2 deletion carriers ^2^.

**RNA-seq.** Male mice, at the age of 4 months, were used for this analysis. The first steps of the analysis, including generation of quality control metrics, alignment of the FASTQ files to the genome, and generation of count files, were all conducted on a high performance Linux cluster using an automated RNA-seq pipeline (version 1.4.2, [https://nf-co.re/rnaseq](https://nf-co.re/rnaseq)) from the nf-core repository ^3^ written in the Nextflow pipeline language ^4^. Singularity containers ^5^ were used to access individual analysis programs from Docker Hub ([https://hub.docker.com/](https://hub.docker.com/)): Hisat2 ^6^ was chosen for alignment to the mouse genome (mm10) while Salmon^7^ was used to generate count files. The following command was used to perform this part of the analysis: All subsequent data analysis was done with custom scripts in R^8^  using R Studio^9^  as the Integrated Development Environment. Handwritten metadata were captured during brain dissections and then manually entered into Excel files, which were subsequently imported into R, combined, and re-shaped with custom scripts using Tidyverse packages ([https://tidyverse.tidyverse.org/articles/paper.html](https://tidyverse.tidyverse.org/articles/paper.html)). Analysis of differential gene expression comparing +/+ and Del/+ was done using a standard DESeq2 ^10^ workflow with the Independent Hypothesis Weighting option ^11^. Figures were generated with custom scripts using the ggplot2 package ^12^. 

**Behavioral assays**

Male mice were sequentially tested for vocalization at P8 and P12 and in a battery of other behavioral tests starting at the age of 1 month. The latter age is considered a “post-pubertal” period, as mice begin to exhibit early signs of puberty around the age of 1 month ^13^. 

We used our published standard behavioral battery that included reciprocal social interaction, novel object approach, olfactory responses to various odorants, spontaneous alternation in T-maze, elevated plus maze (EPM), locomotor activity and thigmotaxis in an inescapable open field, and startle and prepulse inhibition in this order ^14-21^. The experimenter was blinded to genotypes. The order of behavioral assays was based on stress level; behaviors that occur in home cage-like settings were given first (i.e., social interaction, novel object approach, and olfactory responses). As the presence of choices in an apparatus is assumed to impose less stress than in apparatuses with no choice, T-maze and EPM tests were given before an inescapable open field. The most stressful tasks (i.e., startle and PPI) came last. Unless a test is designed to be given on consecutive days (e.g., T-maze with three delays over a period of 3 days), one to two rest days were imposed between different tests to avoid carry over effects ^22^. Mice were additionally tested on working memory at two months, as deficits are more apparent at this age in mice with genetic variants associated with neuropsychiatric disorders ^1, 14, 19^ and toward adulthood in humans with idiopathic ASD ^23, 24^ or a genetic variant ^25^. 

Neonatal USV. The day of birth is defined as Postnatal day 0 (P0).  To control for effects of male breeders on mothers and their pups, male breeder partners were removed from the breeder cage immediately after female pregnancy was found. 

Male pups were tested for vocalization during 5-minute maternal separation at the postnatal day (P) 8 and P12. In mice, P7–P10 corresponds to the term human infants ^26^. 

The apparatus and procedure are detailed in our previous publication ^17^. There was no light inside the testing apparatus. Briefly, pups were separated from their dams and placed in the recording apparatus (26°C) for 5 minutes. Pups’ ultrasonic vocalizations were recorded by UltraSoundGate (Avisoft, Germany) connected to a computer equipped with Avisoft-RECORDER software (Avisoft, Germany). The sampling rate was set at 300 kHz (format, 16 bit). A lower cutoff frequency was set at 15 kHz to reduce background noise outside the relevant frequency band. The frequency window for analysis ranges from 15 kHz to ~ 150 kHz. Call detection was provided by an automatic threshold-based algorithm and a hold time mechanism (hold time: 10 ms). 

 

Using Avisoft SASLab Pro (version 4.40, Avisoft, Germany)), together with Audacity software ([http://audacity.sourceforge.net/download/windows](http://audacity.sourceforge.net/download/windows)), we determined the wave-type of pup vocal calls ^15, 27^. 

We used our classification of call types^27^ 

Complex (Cx): One continuous sound wave with two or more directional pitch changes, each ≥6.25 kHz.

Two syllable (Ts): Two sound waves sequentially emitted with a sudden shift to a higher frequency without a temporal gap.

Frequency steps (Fs): Call that changes into two separate waves simultaneously emitted at different frequencies in the middle with no interruption in time.

Harmonics (Ha): One main sound wave with additional waves at different frequencies surrounding the main wave.

Composite (C): Two sound waves emitted simultaneously at different frequencies.

Hump (Chevron): Inverted-U shaped sound wave, composed of a continuous frequency increase ≥12.5 kHz, followed by a decrease ≥6.25 kHz.

Short (Sh): Sound wave that lasts for <5 msec.

Downward (D): Sound wave with a continuous decrease in pitch that is ≥12.5 kHz, with a terminal dominant frequency at least 6.25 kHz lower than the beginning of the sound wave.

Flat (F): Sound wave that does not ﬂuctuate in frequency more than 3 kHz.

Upward (U): Sound wave with a continuous increase of ≥12.5 kHz, with a terminal dominant frequency at least 6.25 kHz higher than the beginning of the sound wave.

Uncharacterized (Un): Call types that cannot be categorized as any of the above.

Ambiguous (Am): Calls that were close to a call type but did not satisfy at least one criterion of each call classification.

Annotation of vocal call types was routinely performed by two raters independently: an initial inter-rater agreement was > 97%. Cases where the initial annotations were different between the raters were re-evaluated by both raters to reach consensus. By comparing sonograms with the data file automatically generated by Avisoft, false positive signals and false negative calls were eliminated and added, respectively, to the final data file ^15, 27^.  We did not use automatic call detection software, as high false negative rates have been noted ^27, 28^.

Mice were subsequently tested, during the light phase between 1PM and 4PM, for post-pubertal behaviors at 1 months of age. 

Reciprocal social interaction*.*  A stimulus mouse, as an experiment subject, and an unfamiliar C57BL/6NTac mouse (Taconic, Rensselaer, NY) as a stimulus subject, were placed in a home-cage setting (28.5 cm long×17.5 cm wide x 12.5 cm high; ~430 lux) that was novel to both stimulus and test mice and their active reciprocal affiliative social interaction was recorded. Under this experimental condition, mice did not exhibit aggressive behaviors ^16-19, 21^. 

 

Novel object approach*.* This task evaluates the general tendency of the mouse to approach a novel non-mouse object. We used our standard procedure ^16, 17, 21^. The apparatus was a  plastic cage (28.5 cm long×17.5 cm wide x 12.5 cm high; ~430 lux) that contained a modified falcon tube (3 cm diameter × 8.5 cm length).

 

Olfactory responses to social and non-social cues*.* Mice were habituated to the test cage (28.5 cm long × 17.5 cm wide x 12.5 cm high, divided into a 19.5 cm-long compartment and a 9 cm-long compartment with a partition wall with a ~5 cm x ~5 cm opening; ~645 lux) for 15 minutes. Filter paper (Whatman, #3698-325, Maidstone England) was soaked with 10 μl of each odorant and was placed in a 1ml Eppendorf tube, which had 7 holes (1 in the middle and 6 surrounding it) on the cap. The tube was attached to the cage wall with Velcro. The following odors were tested sequentially: water, almond extract (McCormick; Hunt Valley, MD, 1:100), imitation banana extract (McCormick; Hunt Valley, MD, 1:100 dilution), urine of one male C57BL/6NTac, urine of another male C57BL/6NTac, urine of the first C57BL/6NTac mouse, and urine of a male Del/+ mouse.  Mice were given three 2-min trials for each odorant session with a ~10 seconds interval between trials of an odorant and sessions of different odorants. Time mice spent in sniffing at the odorant tube was measured during each 2-min trial. The mouse remained in the test cage until it was tested with all odorants. We used age-matched non-littermate male C57BL/6NTac mice to collect urine a week before testing. The test mouse was never exposed to that urine before testing. Collected urine was kept frozen at -20C till the test day. 

T-maze*.* Spontaneous alternation in the T-maze is a widely used task to measure working memory and memory-based repetitive behavioral tendencies^29^. We used our standard procedure^16, 17, 21^. As working memory deficits are often apparent at 2 months, but not at 1 month of age^14, 19^, we additionally tested mice for this task at 2 months of age. The apparatus was a black Plexiglas T-maze. The three arms were identical in size (30 cm long x 10 cm wide x 20 cm high wall foreach arm; ~914 lux) and were connected by a central area (10 x 10 x 20 cm).

Elevated plus maze*.* This is a widely-used standard mouse task to evaluate anxiety-related behavior ^16, 17, 21^. The apparatus had four arms (30 x 5 cm). Two open arms and two closed arms extended from the center platform (5 x 5 cm), positioned 53 cm above the floor (~914 lux). 

Open field test*.* Horizontal locomotor activity and thigmotaxis in an inescapable open field are well-accepted measures of motor activity and anxiety-related behavior, respectively. Thigmotaxis in an open field is thought to reflect anxiety under a higher level of stress, compared to the elevated plus maze ^30, 31^. Horizontal locomotor activity was analyzed in the entire area (26 cm x 26 cm x 38.5 cm high; ~861 lux; Truscan, Coulbourn Instruments, Allentown, PA, USA). Thigmotaxis was analyzed as time in the marginal area (a 4 cm band extending from the wall) of the entire field^21^.  

 

Startle and Prepulse inhibition*.* We used our standard procedure (S-R LAB, San Diego Instruments, San Diego, CA, USA)^18, 19^. Startle responses were evaluated in a range of stimuli between 75 and 120 dB. Prepulse stimuli were set at 8, 12, 16, 20, and 24 dB above the background noise. The startle sound for PPI was set at 120 dB. 

  

**Computational Analyses** We applied computational analyses to neonatal USVs (see **Fig. S4, Neonatal behaviors**). First, parameter acquisition was done using acoustic signals, as detected by Avisoftware (SASLab Pro, Avisoft Bioacoustics). Because the software often detects noises as USVs (i.e., false positives) and misses real USVs (i.e., false negatives), we visually compared sonograms and acoustic signals generated by Avisoftware to eliminate false positive signals and include false negative signals ^27^. 

Some pups did not emit―or emitted a very few―calls during the entire 5-minute session. Such cases are considered a phenotype and were included in the analysis of call numbers. However, such data cannot be used for analysis of call sequences. Moreover, as cases where a pup emits fewer than 10 total calls skew the analysis of the relative proportion of various call patterns, those cases also were eliminated from the analysis of sequences. Accordingly, we excluded from the analyses of sequences four P8 +/+ cases (Total call numbers = 4, 0, 8, and 0) out of 37 cases, and one P8 Del/+ case (total call number = 8) out of 23 cases; there was no skewed exclusion between the two genotypes (χ2(1)=0.1602, p=0.689). For P12 cases, we excluded thirteen +/+ cases (Total call numbers = 0, 5, 0, 0, 0, 0, 0, 0, 0, 0, 0, 0, and 5) out of 29 cases, and five Del/+ cases out of 15 (Total call numbers = 0, 0, 5, 0, and 0). There was no skewed exclusion between the two genotypes (χ2(1) = 0.5403, p = 0.462).  

This modified dataset was analyzed in two independent ways: categorical distinct call types 

 (52) and quantitative acoustic parameters (see **Fig. S4,** **Parametric acquisition**). For the former method, the sequence of distinct call types was further determined ^15, 27^ and frequently emitted sequences were modelled using Markov models.  

We ran least absolute shrinkage and selection operator (Lasso) regression model (**Fig. S4**, **Predictive models for social behaviors**, **Lasso regression model 1**), as it extracts a small number of predictive features from all acoustic features, including acoustic parameters, number and ratios of distinct call types and number and probabilities of distinct two-call transitions (i.e., sequences).  The dependent variables were affiliative, active social interaction, its habituation from Session 1 to 2, olfactory responses to the first urine smell at Trial 1 and its habituation from Trial 1 to 3. As not all mice survived to be tested at the age of 1 month, the sample sizes used for analysis of post-pubertal behaviors were less than those used for analysis of neonatal vocalization (see **Fig. 2** legend). 

Call probabilities were calculated separately for +/+ and Del/+ pups.  Due to the sparsity of data, the Perks prior (1/N pseudo count value) was included in analysis ^32^. Shannon entropy analysis was performed on call-type sequences of up to length four to determine the overall structure in the call sequences. Statistical analysis of entropy data was carried out using a mixed-effects linear model with entropy being modelled as a function of both group and level and each pup having its own baseline entropy. 

Sparse partial least squares-discriminant analysis (sPLS-DA) and Markov modeling were performed on call type sequences of length two.  As the “u-shape” call was unique to the P12 data, it was excluded from analysis. sPLS-DA analysis used call transition probabilities of each individual mouse, so that individual variation in call transition can be identified. The difference in transition probabilities between the +/+ and DEL/+ was calculated using Jensen-Shannon divergence metric for the distribution of call types. The transition probability matrix (TPM) from the Markov model was used based on the fact that each row of a TPM is a probability distribution by itself. For Markov modeling, we intended to identify frequently emitted two call transitions for each genotype and thus call transition counts were pooled within each genotype to compute call transition probabilities. Pseudo counts were added to avoid zero probabilities. The probabilities  (*Pi*) were computed using the following formula:  where *Calli* represent the count of a two-call sequence, α is the pseudo count value, N is the sum of all two-call sequence counts that start with the same call type being calculated (e.g., A->A, A->C, A->Cx, etc) and *d* is the number of all possible two-call combinations of 11 call types (11^2=121); “Uncharacterized” calls (Un) were included in Ambiguous cases, because neither case belongs to any of the preset call type. .  

In parallel to Markov models, specific two call-sequences, among all 121 possible two call-sequences, that predict genotype were further identified by Lasso regression models and its predictive capacity was evaluated by Random Forest (see **Fig. S4**, **Predictive models for genotype, Lasso regression model 2** and **Random forest 1**). We took the variables selected by LASSO with deviance ratio=0.5 to avoid over-fitting and ran random forests 20 times on 2-fold cross-validation using R package random Forest.

The two-call sequences that were selected by Markov models and Lasso 2/Random Forest 1 were used to construct linear regression models to predict post-pubertal social behaviors at 1 month of age (see **Fig. S4**, **Predictive models for social behaviors, Linear regression model**). The dependent variables were affiliative, active social interaction, its habituation from the first to the last session (Sessions 1 to 2), olfactory responses to a urine smell and its habituation from the first to last session (Sessions 1 to Session 3). 

Twenty-three acoustic parameters were analyzed by Random Forest (Python3.6.5, scikit-learn 0.19.1) on a 10-fold cross-validation (GridSearch, 3≦estimators≦20, 3≦depth≦20) to determine their predictability for genotype (see **Fig. S4**, **Predictive models for genotype, Random forest 2**). 

**Code availability**

All computer programs and data are available upon request.

**References**

1.	Hiroi N. Critical Reappraisal of Mechanistic Links of Copy Number Variants to Dimensional Constructs of Neuropsychiatric Disorders in Mouse Models. *Psychiatry and Clinical Neurosciences* 2018; **72**(5)**:** 301-321.

2.	Hudac CM, Bove J, Barber S, Duyzend M, Wallace A, Martin CL *et al.* Evaluating heterogeneity in ASD symptomatology, cognitive ability, and adaptive functioning among 16p11.2 CNV carriers. *Autism Res* 2020; **doi: 10.1002/aur.2332. Online ahead of print.**

3.	Ewels PA, Peltzer A, Fillinger S, Patel H, Alneberg J, Wilm A *et al.* The nf-core framework for community-curated bioinformatics pipelines. *Nat Biotechnol* 2020; **38**(3)**:** 276-278.

4.	Di Tommaso P, Chatzou M, Floden EW, Barja PP, Palumbo E, Notredame C. Nextflow enables reproducible computational workflows. *Nat Biotechnol* 2017; **35**(4)**:** 316-319.

5.	Kurtzer GM, Sochat V, Bauer MW. Singularity: Scientific containers for mobility of compute. *PLoS One* 2017; **12**(5)**:** e0177459.

6.	Kim D, Langmead B, Salzberg SL. HISAT: a fast spliced aligner with low memory requirements. *Nat Methods* 2015; **12**(4)**:** 357-360.

7.	Patro R, Duggal G, Love MI, Irizarry RA, Kingsford C. Salmon provides fast and bias-aware quantification of transcript expression. *Nat Methods* 2017; **14**(4)**:** 417-419.

8.	Team TRDC. R: A langiage and environment for statistical computing. [*https://wwwyumpucom/en/document/view/6853895/r-a-language-and-environment-for-statistical-computing*](https://wwwyumpucom/en/document/view/6853895/r-a-language-and-environment-for-statistical-computing) 2019.

9.	R Idf. [http://www.rstudio.com/](http://www.rstudio.com/).

10.	Love MI, Huber W, Anders S. Moderated estimation of fold change and dispersion for RNA-seq data with DESeq2. *Genome Biol* 2014; **15**(12)**:** 550.

11.	Ignatiadis N, Klaus B, Zaugg JB, Huber W. Data-driven hypothesis weighting increases detection power in genome-scale multiple testing. *Nat Methods* 2016; **13**(7)**:** 577-580.

12.	Wickham H. ggplot2 : Elegant Graphics for Data Analysis.  *Use R!,*, 2nd edn. Springer International Publishing : Imprint: Springer,: Cham, 2016, pp 1 online resource (XVI, 260 pages 232 illustrations, 140 illustrations in color.

13.	Bronson FH, Dagg CP, Snell GD. Reproduction. In: Green EL (ed). *Biology of the Laboratory Mouse*, Second Edition edn. Dover Publications, Inc.: New York, 2007, p Oneline publication.

14.	Boku S, Izumi T, Abe S, Takahashi T, Nishi A, Nomaru H *et al.* Copy number elevation of 22q11.2 genes arrests the developmental maturation of working memory capacity and adult neurogenesis. *Molecular Psychiatry* 2018; **23**(4)**:** 985-992.

15.	Takahashi T, Okabe S, Broin PO, Nishi A, Ye K, Beckert MV *et al.* Structure and function of neonatal social communication in a genetic mouse model of autism. *Mol Psychiatry* 2016; **21**(9)**:** 1208-1214.

16.	Harper KM, Hiramoto T, Tanigaki K, Kang G, Suzuki G, Trimble W *et al.* Alterations of social interaction through genetic and environmental manipulation of the 22q11.2 gene Sept5 in the mouse brain. *Human Molecular Genetics* 2012; **21**(15)**:** 3489-3499.

17.	Hiramoto T, Kang G, Suzuki G, Satoh Y, Kucherlapati R, Watanabe Y *et al.* Tbx1: identification of a 22q11.2 gene as a risk factor for autism spectrum disorder in a mouse model. *Hum Mol Genet* 2011; **20**(24)**:** 4775-4785.

18.	Suzuki G, Harper KM, Hiramoto T, Sawamura T, Lee M, Kang G *et al.* Sept5 deficiency exerts pleiotropic influence on affective behaviors and cognitive functions in mice. *Human Molecular Genetics* 2009; **18**(9)**:** 1652-1660.

19.	Suzuki G, Harper KM, Hiramoto T, Funke B, Lee M, Kang G *et al.* Over-expression of a human chromosome 22q11.2 segment including TXNRD2, COMT and ARVCF developmentally affects incentive learning and working memory in mice. *Human Molecular Genetics* 2009; **18**(20)**:** 3914-3925.

20.	Hiroi N, Zhu H, Lee M, Funke B, Arai M, Itokawa M *et al.* A 200-kb region of human chromosome 22q11.2 confers antipsychotic-responsive behavioral abnormalities in mice. *Proceedings of the National Academy of Sciences of the United States of America* 2005; **102**(52)**:** 19132-19137.

21.	Yamauchi T, Kang G, Hiroi N. Heterozygosity of murine Crkl does not recapitulate behavioral dimensions of human 22q11.2 hemizygosity. *Genes Brain Behav* 2020**:** e12719.

22.	Paylor R, Spencer CM, Yuva-Paylor LA, Pieke-Dahl S. The use of behavioral test batteries, II: effect of test interval. *Physiol Behav* 2006; **87**(1)**:** 95-102.

23.	Luna B, Doll SK, Hegedus SJ, Minshew NJ, Sweeney JA. Maturation of executive function in autism. *Biol Psychiatry* 2007; **61**(4)**:** 474-481.

24.	Rosenthal M, Wallace GL, Lawson R, Wills MC, Dixon E, Yerys BE *et al.* Impairments in real-world executive function increase from childhood to adolescence in autism spectrum disorders. *Neuropsychology* 2013; **27**(1)**:** 13-18.

25.	Dumontheil I, Roggeman C, Ziermans T, Peyrard-Janvid M, Matsson H, Kere J *et al.* Influence of the COMT genotype on working memory and brain activity changes during development. *Biol Psychiatry* 2011; **70**(3)**:** 222-229.

26.	Semple BD, Blomgren K, Gimlin K, Ferriero DM, Noble-Haeusslein LJ. Brain development in rodents and humans: Identifying benchmarks of maturation and vulnerability to injury across species. *Prog Neurobiol* 2013; **106-107:** 1-16.

27.	Ó Broin PB, M.V.; Takahashi, T.; Izumi, T.; Ye, K.; Kang, K.; Pouso, P.; Topolski, M.; Pena, J.L.; Hiroi, N. Computational Analysis of Neonatal Mouse Ultrasonic Vocalization. *Current Protocols in Mouse Biology* 2018; **8**((2))**:** e46.

28.	Binder M, Nolan SO, Lugo JN. A comparison of the Avisoft (v.5.2) and MATLAB Mouse Song Analyzer (v.1.3) vocalization analysis systems in C57BL/6, Fmr1-FVB.129, NS-Pten-FVB, and 129 mice. *J Neurosci Methods* 2020**:** 108913.

29.	Lalonde R. The neurobiological basis of spontaneous alternation. *Neurosci Biobehav Rev* 2002; **26**(1)**:** 91-104.

30.	Zhu H, Lee M, Agatsuma S, Hiroi N. Pleiotropic impact of constitutive fosB inactivation on nicotine-induced behavioral alterations and stress-related traits in mice. *Human Molecular Genetics* 2007; **16**(7)**:** 820-836.

31.	Misslin R, Herzog F, Koch B, Ropartz P. Effects of isolation, handling and novelty on the pituitary--adrenal response in the mouse. *Psychoneuroendocrinology* 1982; **7**(2-3)**:** 217-221.

32.	Perks W. Some observations on inverse probability including a new indifference rule. . *J Inst Actuaries* 1947; **73:** 285-334.

**Supplemental Figure legends**

**Figure S1**. Generation and characterization of a mouse model of human 16p11.2 hemizygosity. **A**) Design of gene targeting. **B**) RNA-seq determined expression levels of genes encoded in the mouse homolog of human 16p11.2 locus. Genes from Mapk3 to Spn were significantly reduced in expression levels (red dots) in various brain regions of Del/+ mice compared to +/+ mice. Some genes (see grey dots) did not show statistically significant reductions in Del/+ mice as compared to +/+ mice due to large variance. Those genes differed from one anatomical region to another. Male mice were sacrificed for analysis at approximately 4 months of age. +/+: Cortex, *N* = 6; Dentate Gyrus, *N* = 4; Hippocampal CA1/CA3, *N* = 6; Medial Prefrontal Cortex, *N* = 6; Striatum, *N* = 6. Del/+: Cortex, *N* = 6; Dentate Gyrus, *N* = 3; Hippocampal CA1/CA3, *N* = 5; Medial Prefrontal cortex, *N* = 6; Striatum, *N* = 5.

**Figure S2.** Post-pubertal behaviors (Mean±SEM) at 1 month of age. **A**) Novel object approach. +/+ mice and Del/+ mice were indistinguishable (Genotype, F(1,18) = 0.061, p = 0.807; Genotype x Session, F(1,18) = 0.000068, p = 0.994). +/+, n = 12; Del/+, n = 8. **B,C**) Elevated plus maze. +/+ mice and Del/+ mice were indistinguishable in the relative time spent in open arms (**B**, U=30, p=0.3104) and frequency to visit open arms (**C**, U=28.5, p=0.2446). +/+, n = 12; Del/+, n = 7. **D**) Startle to acoustic stimuli. +/+ mice and Del/+ mice were indistinguishable (75dB, U=80.50, p=0.6681; 80dB, U=67.50, p=0.2998; 85dB, U=70.50, p=0.3709; 90dB, U=73.0, p=0.4365; 95dB, U=59.50, p=0.1558; 100dB, U=75.50, p=0.5085; 105dB, U=65.50, p=0.2575; 110dB, U=69.50, p=0.3462; 120dB, U=83.00, p=0.7557). +/+, n = 20; Del/+, n = 9. **E**) Acoustic prepulse inhibition. +/+ mice and Del/+ mice were indistinguishable (69dB, U=84, p=0.7992; 73dB, U=83, p=0.7637; 77dB, U=75, p=0.5014; 81dB, U=83, p=0.7637; 85dB, U=83, p=0.7637; 89dB, U=75, p=0.5014). +/+, n = 20; Del/+, n = 9. Each prepulse stimulus was presented against a 65 dB background. **F**) Spontaneous alternation. +/+ mice and Del/+ mice were indistinguishable (0 sec, U=62, p=0.7613; 30s, U=71.50, p>0.9999).  +/+, n = 18; Del/+, n = 8. Open field. +/+ mice and Del/+ mice were indistinguishable in **G**) distance traveled (Genotype, F(1,26) = 0.633, p = 0.443; Genotype x Time, F(5,130) = 0.570, p = 0.723) and **H**) time spent in the margin area (5min, U=84, p=0.9519; 10mim, U=67.5, p=0.3892; 15min, U=77.5, p=0.7075; 20min, U=66, p=0.3568; 25min, U=51, p=0.0929; 30min, U=46, p=0.0536). +/+, n = 19; Del/+, n = 9. 

**Figure S3**. The body weights of +/+ and Del/+ mice at 1 and 2 months of age. Body weights increased from 1 to 2 months of age in both genotypes, but the body weights of Del/+ mice were consistently lower than those of +/+ mice at the both developmental time points (Genotype, F(1, 21.223) = 6.334, p=0.020; Age, F(1, 16.522) = 301.011, p<0.001; Genotype x Age, F(1, 16.522) = 2.808, p=0.113). 1 month, +/+, n = 15, Del/+, n = 8. +/+; 2 months, n = 13, Del/+, n = 5.

**Figure S4**. The general strategy to analyze the neonatal USVs. Following parameter acquisition and classification, linear regression and lasso regression model were used to predict social behaviors at 1 month of age from dimensions of neonatal vocalization. Genotype was incorporated as a variable in green lines.

**Figure S5**. Selection of call features to predict genotype (see **Lasso regression model 2,** **Fig. S4**). Cutoff was set at 0.5 of fraction deviance explained (see light grey vertical broken line), and only the variables used in Random Forest are labeled. 

**Figure S6**. Correlations of post-pubertal social behaviors with call features identified by Lasso regression model 1, Markov models and Lasso regression model 2. This analysis used only those mice that were tested for both neonatal vocalization and social or olfactory responses at 1 month of age. Mice that emitted less than 10 calls were excluded from analysis, as the relative proportions of various call sequences cannot be reliably estimated. Predictors either genotype (+/+ and Del/+) are grouped. A, B: +/+. n=11; Del/+, n=8. C,D,  +/+. n=16; Del/+, n=8.

Variables selected by Lasso 1 model (see **Fig 2**; **Fig S4**) that had correlation coefficients of P<0.05 with social behaviors. This model selected variables that predict social behaviors. All variables were significant for goodness of fit, as assessed by Analysis of Deviance (AoV). Multiple comparisons were adjusted by Benjamini-Hochberg correction with a false discovery rate of 0.05. 

2

 **Table S2** 

Confusion matrix

Accuracy	0.681

Sensitivity	0.5000

Specificity	0.7917

Confusion matrix of a 2-class classifier by Random Forest (see **Fig. S4**, **Random forest 1**) to predict genotypes from call features. Del/+ was regarded as positive reference.

**Table S3**

Variables selected by Lasso model 2 that had correlation coefficients (P<0.05) with social behaviors (see **Fig. S4** and **S5**). This model selected variables that predict genotype. All variables were significant for goodness of fit, as assessed by Analysis of Deviance (AoD). Multiple comparisons were adjusted by Benjamini-Hochberg correction with a false discovery rate of 0.05. MaFmi: MaF (maximum frequency), frequency (kHz) at which the amplitude of the spectrum crosses the threshold on the descending slope on the frequency scale. mi (minimum parameter of the entire call), minimum of each parameter of all spectra within an entire call.

**Table S4** 

**Table S5** 

Variables selected by Markov models (see **Fig. 3C**) that had correlation coefficients (P<0.05) with social behaviors (see **Fig. S4**). The Markov model selected call transitions that are most frequently emitted by either genotype. All variables were significant for goodness of fit, as assessed by Analysis of Deviance. Multiple comparisons were adjusted by Benjamini-Hochberg correction with a false discovery rate of 0.05. 

Confusion matrix

Accuracy	0.5642

Sensitivity	0.4946

Specificity	0.6338

**Table S6** 

Confusion matrix of the classifier by Random Forest of a 2-class classifier to predict genotypes from acoustic parameters for each call. Del/+ was regarded as positive (see **Fig. S4**, **Random Forest 2**).
